# Characterization of the prion protein gene in axis deer (*Axis axis*) and implications for susceptibility to chronic wasting disease

**DOI:** 10.1080/19336896.2021.1910177

**Published:** 2021-04-09

**Authors:** Matthew J. Buchholz, Emily A. Wright, Blake A. Grisham, Robert D. Bradley, Thomas L. Arsuffi, Warren C. Conway

**Affiliations:** a Department of Natural Resources Management, Texas Tech University,Lubbock, TX USA; b Department of Biological Sciences, Texas Tech University, Lubbock, TX USA; c Natural Science Research Laboratory, Museum of Texas Tech University, Lubbock, TX USA; d Llano River Field Station, Texas Tech University, Junction, TX USA

**Keywords:** *Axis axis*, axis deer, chital, chronic wasting disease, prion protein, *prnp*, prp

## Abstract

Axis deer (*Axis axis*) occur both in captivity and free-ranging populations in portions of North America, but to-date, no data exist pertaining to the species’ susceptibility to CWD. We sequenced the prion protein gene (*PRNP*) from axis deer. We then compared axis deer PrP^C^ sequences and amino acid polymorphisms to those of CWD susceptible species. A single *PRNP* allele with no evidence of intraspecies variation was identified in axis deer that indicates axis deer *PRNP* is most similar to North American elk (*Cervus canadensis*) *PRNP*. Therefore, axis deer may be susceptible to CWD. We recommend proactively increasing CWD surveillance for axis deer, particularly where CWD has been detected and axis deer are sympatric with native North American CWD susceptible species.

## Introduction

Chronic wasting disease (CWD) is a fatal, transmissible spongiform encephalopathy (TSE) of cervids that belongs to the greater classification of TSE/prion diseases [e.g. bovine spongiform encephalopathy in cattle (*Bos taurus*), scrapie in sheep (*Ovis aries*) and goats (*Capra hircus*), feline spongiform encephalopathy in felids, and Creutzfeldt-Jakob disease in humans (*Homo sapiens*)], which is caused by the aggregation of a misfolded isoform (PrP^CWD^) of the cellular prion protein (PrP^C^) [1,2]. The cellular prion protein is encoded for in its entirety by the third exon of the prion protein gene (*PRNP*) [1]. Chronic wasting disease was first documented in captive mule deer (*Odocoileus hemionus*) in Colorado in the 1960s [2], and has since been detected in free–ranging and captive cervid populations in 26 US states and 3 Canadian provinces, as well as internationally in Finland, Norway, Sweden, and South Korea [3–6]. Cases of CWD have been detected in other native North American species, including white–tailed deer (*Odocoileus virginianus*), North American elk (*Cervus canadensis*), and moose (*Alces alces*), as well as species that are not native, but have been introduced, to North America including reindeer (*Rangifer tarandus*), sika deer (*C.* *nippon*), red deer (*C.* *elaphus*), Reeve’s muntjac (*Muntiacus reevesi*), and fallow deer (*Dama dama*) [3–8]. The expanding distribution, movement of, and increasing number of, known susceptible species is a concern for wildlife stakeholders worldwide, and efforts to limit the spread of CWD are extensive.

Many studies have focused upon assessing CWD susceptibility in cervids by sequencing *PRNP* and identifying amino acid substitutions in PrP^C^. For example, amino acid substitutions such as Q95H, G96S, A116G, and Q226K in white-tailed deer, S225F in mule deer, and M132L in elk are linked to reduced CWD prevalence and prolonged incubation periods/life expectancy post–CWD infection within the proportion of those species with those substitutions [1,9–11]. The potential for interspecific transmission of CWD between individuals of different cervid species with different *PRNP* genotypes and PrP^C^ variants is documented in mouse models, and data suggests CWD susceptibility is not limited to species within Cervidae [12–15]. Phylogenetic analyses and challenge studies indicate species beyond Cervidae, including pronghorn (*Antilocapra americana*), bighorn sheep (*Ovis canadensis*), mountain goat (*Oreamnos americanus*), and squirrel monkeys (*Saimiri sciureus*) may be susceptible to infection by the CWD prion and CWD susceptibility is not monophyletic within Cervidae [13,16,17]. The various lines of evidence suggest many, if not all, members of Cervidae are likely susceptible to CWD, including those that naturally occur outside of North America [18,19].

**Figure 1.**
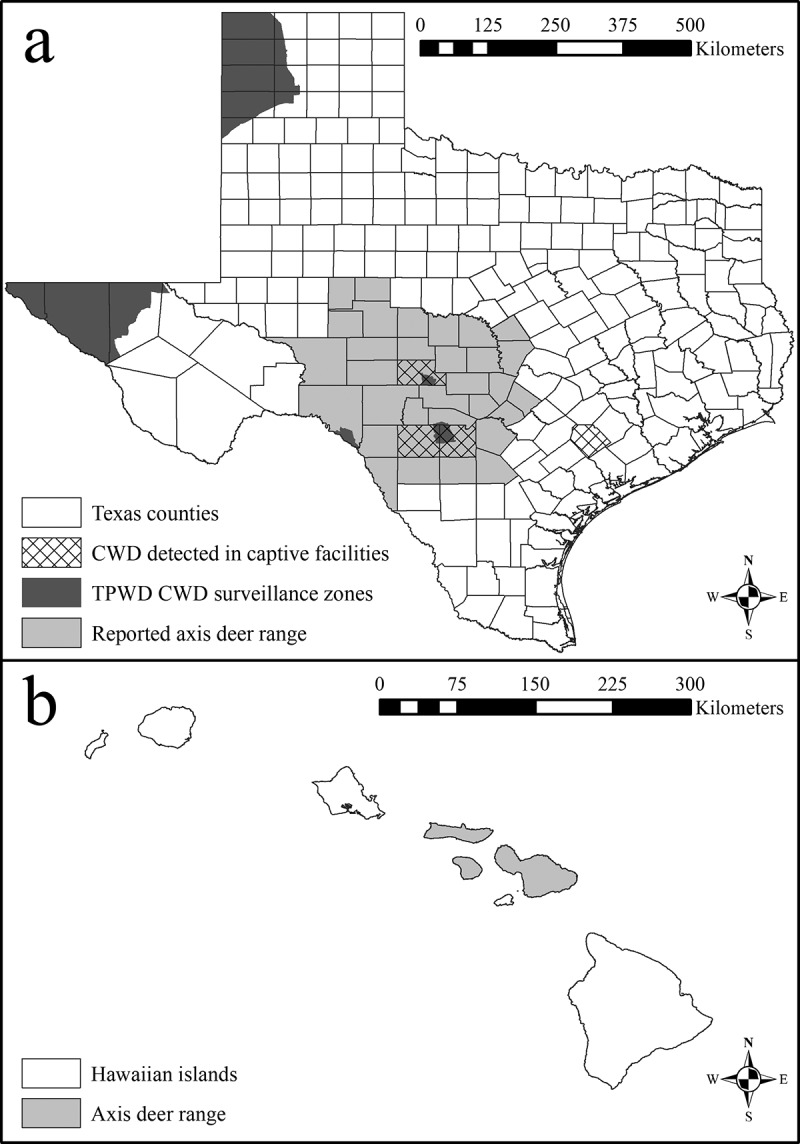
Reported county-level distribution of free-ranging axis deer (*Axis axis*) in Texas (a) and islands where free-range axis deer occur in Hawaii (b). The Texas map includes locations of the chronic wasting disease (CWD) surveillance zones established by Texas Parks and Wildlife Department (TPWD) in response to CWD having been detected in free-ranging and captive cervids

Axis deer (*Axis axis*) are cervids native to the Indian subcontinent, but have been introduced to localities across North America. Although most introduced axis deer are captive in high-fenced ranches, large, free-ranging axis deer populations exist in Hawaii and Texas outside of properties enclosed by high-fences (Figure 1). Axis deer were first introduced to the Hawaiian island of Molokai in 1868, and then spread to several islands via translocations, and efforts to eradicate axis deer in Hawaii have been ineffective [20]. Axis deer were introduced to Texas in 1932 and following intentional emancipation and unintentional escape from confined ranches are now the most abundant free–ranging exotic cervid in Texas, and coexist with native cervid species. Regional biologists with Texas Parks and Wildlife Department (TPWD) speculate that the population is growing in both size and geographic distribution [21–23]. Self-sustaining, naturally reproducing, and free-ranging populations of axis deer exist in 3 of the 5 CWD surveillance zones established by TPWD as well as near or in CWD-positive captive deer facilities in Texas (Figure 1). Currently, no information exists pertaining to the risk of axis deer contracting and spreading CWD, and only 187 axis deer samples have been tested for CWD in Texas (all 187 have been negative). Comparatively, > 100,000 CWD tests have been administered to white-tailed deer and mule deer in Texas since 2012 [24]. Axis deer are legally classified as non-susceptible to CWD in Texas; however, the classification is based on the lack of a positive CWD test result, and not on an investigation into the biological susceptibility to infection with the CWD prion.

The goal of this study was to assess the susceptibility of axis deer to CWD by characterizing *PRNP* exon 3. Given that axis deer are closely related to the genus *Cervus* in the phylogeny of Cervidae [25], we hypothesized that axis deer *PRNP* would be similar to *Cervus* spp. including elk, red deer, and sika deer. Our objectives were to 1) identify and compare any amino acid polymorphisms within axis deer PrP^C^ to known polymorphisms in other species, particularly, polymorphisms that are known to impact CWD susceptibility, and 2) conduct phylogenetic analyses to assess the phylogenetic relationship of PRNP among axis deer and known CWD susceptible species.

## Results

**Figure 2.**
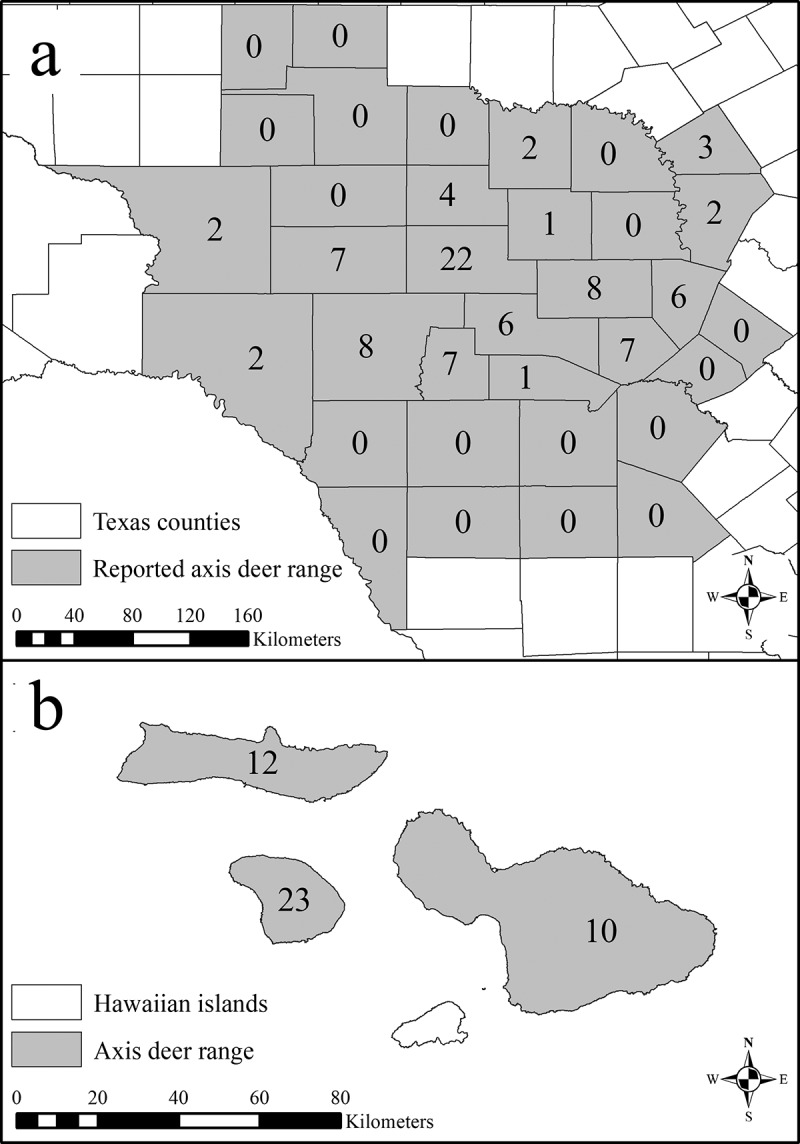
Spatial distribution of the free-ranging axis deer (*Axis axis*) sequenced for *PRNP* exon 3 for this study and the reported ranges of free-ranging axis deer in Texas (a) and Hawaii (b)

*PRNP* exon 3 (771 bp) was sequenced from 133 axis deer (88 from 16 Texas counties and 45 from 3 Hawaiian islands; Figure 2). A single genotype, lacking individual variation, was detected from all populations. Sequences from all 133 individuals have been submitted to the GenBank databases under accession numbers MT996365–MT996497.

### Characterization of PRNP exon 3

In comparison to the consensus cervid PrP^C^ amino acid sequence [1], axis deer sequenced in this study possess 2 amino acid substitutions: Q226E and F249V. The amino acid substitution at residue 226 is consistent with other Old-World cervids of the subfamily *Cervinae* [1]. However, the amino acid substitution at residue 249 has not been identified in any cervid species. All other components of axis deer PRNP were consistent with cervid *PRNP*, including the amino acid makeup of the terminal signals and 5 peptide repeats with 3 octapeptides of PHGGGWGQ flanked by 2 nonapeptides of P(Q/H)GGGGWGQ.

### Phylogenetic analyses

**Figure 3.**
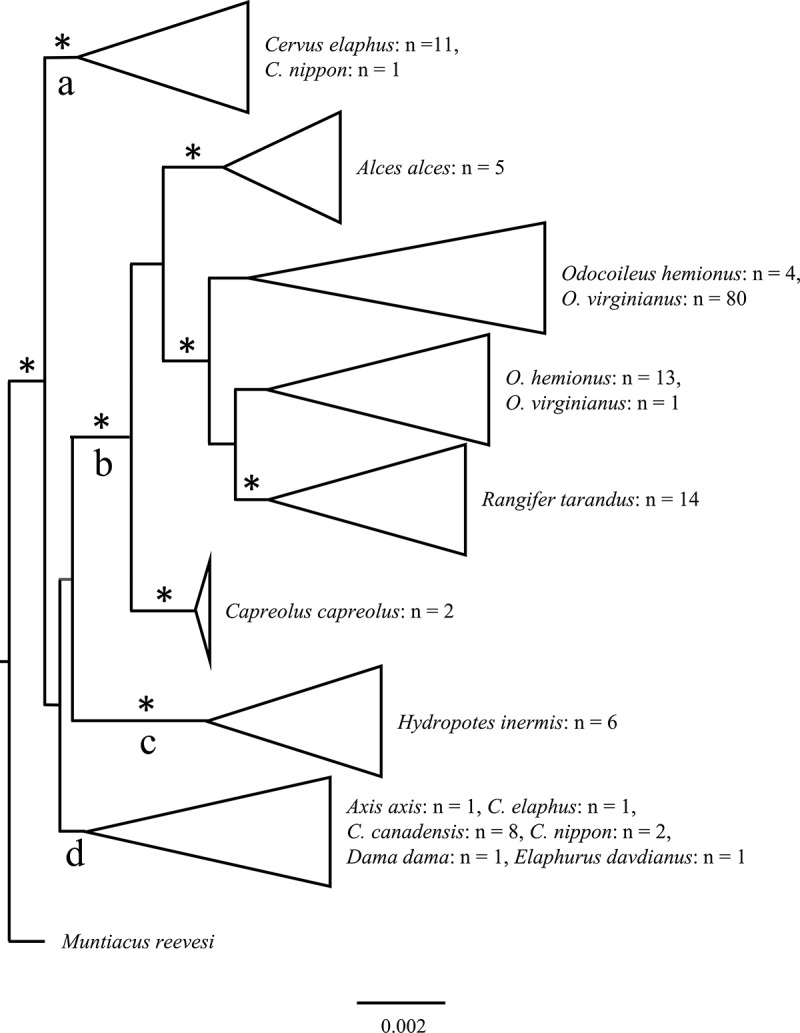
Phylogenetic tree generated from prion protein gene (*PRNP*) sequences from Cervidae with Bayesian inference analyses (MrBayes 3.2) [37]. Nodal support (posterior probability values ≥ 0.95) [38] is indicated by an asterisk above the node. Clades are identified by letters below the node that contains the identified clade. Although there was a lack of continued support beyond the basal node, 8 groups composed of individual species or closely related species in the larger Cervidae phylogeny [24] were distinguishable from the analyses

Support for differentiation of *PRNP* in Cervidae was limited to clade a (Figure 3). There were 8 groups composed of individual species or at least 2 closely related species in the larger Cervidae phylogeny [25]. Although there was no continued support for the differentiation of *PRNP* in Cervidae beyond clade a, axis deer grouped most closely with members of the *Cervus* genus including elk, red deer, and sika deer in clade d. Additionally, although there was some terminal support for clades b & c within the subfamily *Capreolinae*, there was no such support between axis deer and the rest of the subfamily *Cervinae* in clade d (Figure 3).

## Discussion

The entire *PRNP* exon 3 was sequenced from free-ranging axis deer in Texas and Hawaii. Out of the other species with available *PRNP* sequences on GenBank, axis deer were most similar to elk in PrP^C^ amino acid sequence. The complete lack of *PRNP* sequence diversity among all axis deer individuals sequenced in this study from Texas and Hawaii was unexpected given known *PRNP* diversity in other species closely related to axis deer (e.g., 6/771 nucleotide sites are variable in elk, 11/771 in red deer, and 4/771 in sika deer) [1] and the number of individuals sequenced. However, the highly conserved nature of *PRNP* within and among taxa may contribute to this finding [1,26]. Furthermore, a previous assessment of *PRNP* found *PRNP* to be monomorphic in roe deer (*Capreolus capreolus*, n = 297) and fallow deer (n = 66) [19].

Axis deer displayed none of the amino acid substitutions known to result in reduced susceptibility to CWD between individuals with the substitution compared to those without of other cervid species, but did show two amino acid substitutions. The first amino acid substitution (Q226E) is consistent with the majority of known sequences from the subfamily *Cervinae* which may confer decreased susceptibility to CWD in elk and other species in *Cervinae* compared to species in the subfamily *Capreolinae* [1,27]. The second amino acid substitution (F249V) previously has not been identified in any other Cervidae species. Given the introduced nature of axis deer populations in Texas and Hawaii, it is possible that other unidentified nucleotide substitutions and amino acid polymorphisms exist in PrP^C^ in axis deer in their native range that are not represented in free-ranging populations in Texas and Hawaii as an artefact of a founder effect of limited founding individuals of these populations. However, the lack of any *PRNP* sequence diversity within axis deer in Texas and Hawaii, and common amino acid sequence of PrP^C^ to species that are susceptible to CWD (e.g., elk) suggests axis deer in Texas and Hawaii may be susceptible to CWD.

**Figure 4.**
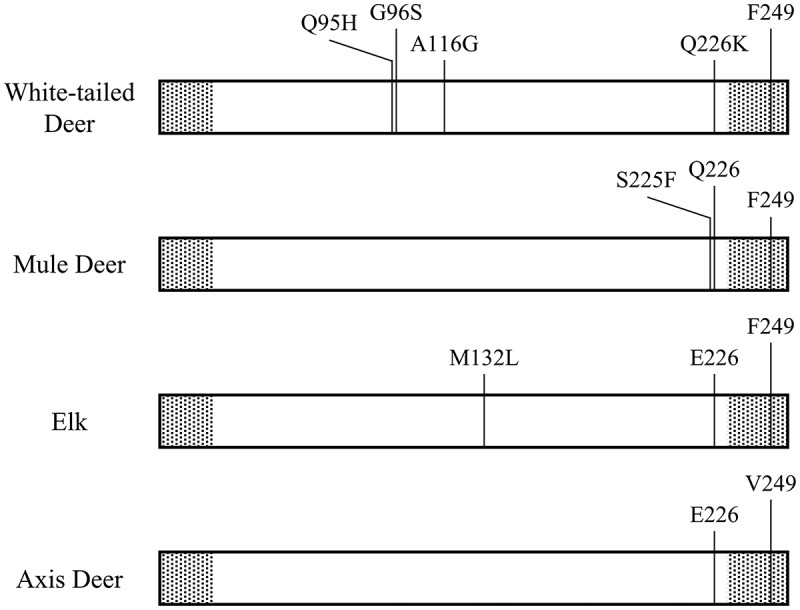
Polymorphic amino acid residues that have been linked to Chronic Wasting Disease susceptibility and the Cervid PrP^C^ variant differentiating amino acid residue (residue 226) in white–tailed deer (*Odocoileus virginianus*), mule deer (*O. hemionus*), and North American Elk (*Cervus canadensis*) [1] in comparison to the axis deer (*Axis axis*) amino acid sequence identified in this study. The substitutions shown convey some level of reduced susceptibility in the form of longer incubation times and reduced prevalence in the proportion of each respective species that possess the substitution genotype compared to the proportion that possess the wild type genotype [1,9–11]. Axis deer possess the wild type genotype at all the polymorphic sites listed for the other species, the elk PrP^C^ variant, and a previously unidentified substitution at residue 249. The shaded areas depict the terminal sequences removed during post-translational editing of PrP^C^ [27]

The identification of a novel amino acid substitution at residue 249 in axis deer poses the question of whether this substitution has an effect on the susceptibility of PrP^C^ to misfolding into PrP^CWD^. However, the mature protein (i.e., the portion of PrP^C^ that misfolds after contact with PrP^CWD^) that results after the terminal signals are removed during post-translational editing is from amino acid residues 23–231 [28]. Residue 249 is located in the C-terminal signal and therefore is outside of the mature protein (Figure 4), and is unlikely to have an effect on the conformation of mature PrP^C^ or PrP^CWD^. Similar findings have been documented with amino acid substitutions outside of the mature protein in other cervid species [29].

Axis deer have the amino acid substitution Q226E that is characteristic of elk and other members of Cervinae from white-tailed deer and other members of Capreolinae [1]. Furthermore, after the amino acid substitution at residue 249 in axis deer is removed during post-translational editing of PrP^C^, axis deer have the same amino acid sequence as the wild type genotype of elk (M132; ~63% of elk; Figure 4), which also is the elk genotype that is most susceptible to CWD [1,10,28]. There was a lack of continued support for differentiation of *PRNP* between known cervid sequences, including the sequence obtained for axis deer from this study. The lack of significant differentiation of *PRNP* within Cervidae supports that many, if not all, cervids potentially are susceptible to CWD, including axis deer, because their ensuing PrP^C^ amino acid sequences likely do not have sufficient diversity to prevent misfolding [30], making them susceptible to CWD infection.

Definitive evidence such as a positive ELISA or IHC test or a protein misfolding cyclic amplification (PMCA) study of axis deer PrP^C^ is needed to conclusively demonstrate axis deer are capable of contracting CWD. However, this research in combination with other work indicating a wider range of susceptible species than previously known [13,19], suggests axis deer in Texas and Hawaii may be susceptible to CWD, and should be managed as an at-risk species for CWD through the implementation of more formalized CWD surveillance. The proactive establishment of a CWD testing programme in axis deer wherever CWD has been detected in other species may be justified. Areas where axis deer coexist with native North American, CWD susceptible species, namely white-tailed deer, may also be justified. In Texas, coordinated CWD testing for axis deer might be important within previously established CWD surveillance and containment zones, followed by surveillance in areas where CWD may be a concern but not yet detected. Due to geographic isolation from areas where CWD occurs, axis deer populations in Hawaii are likely not high risk for CWD occurrence. However, awareness of the possibility and care should be taken to ensure CWD-infected material/animals does not arrive on the islands.

## Materials and Methods

### Tissue sample collection

Tissue samples were collected from 133 axis deer (88 from 16 Texas counties and 45 from 3 Hawaiian islands; Figure 2) from 2017–2020. Ear clip (2 cm^2^) and muscle tissues (2 cm^3^) were obtained by hunter-harvests, live capture with drop nets, and opportunistic sampling from roadkill specimens. Tissues (ear clip and muscle) from Texas were immediately frozen after collection and stored at −20°C whereas tissues from Hawaii were stored and shipped in RNA*later* (Invitrogen, Waltham, MA, USA) and immediately stored at −20°C upon acquisition. All remaining tissue samples were catalogued and archived at the Natural Science Research Laboratory at the Museum of Texas Tech University. Tissue samples were collected following methodology outlined in guidelines of the American Society of Mammalogists [31] and approved by the Texas Tech University Institutional Animal Care and Use Committee permit #17030–40.

### DNA amplification and sequencing

Genomic DNA (gDNA) was isolated from 0.1 g piece of ear clip, and muscle tissue when available, using the Qiagen DNeasy kit (Qiagen Inc., Valencia, CA, USA). The full length of *PRNP* exon 3 (771 bp) and up to 53 bp of the 5ʹ flanking sequence and 83 bp of the 3ʹ flanking sequence was amplified using polymerase chain reaction methods (PCR) [32] with the amplification primers MD582F and MD1479RC [9]. All PCR reactions followed the standard HotStarTaq (Qiagen Inc., Valencia, CA, USA) protocol: 25 µL reactions containing 30 ng gDNA, 12.5 µL HotStarTaq premix, 0.6 µL of each 10 µM primer, and 8.3 µL of distilled water (ddH_2_O). The thermal profile was as follows: hot start of 80°C, initial denaturation at 95°C for 2 min, followed by 34 cycles of denaturation at 95°C for 30 s, annealing (range: 53-54°C) for 45 s, and extension at 73°C for 1 min, with a final extension at 73°C for 15 min.

PCR products were purified with ExoSAP-IT PCR Product Cleanup (Applied Biosystems, Foster City, CA, USA). Cycle sequence reactions were carried out using BigDye Terminator v3.1 (Applied Biosystems, Foster City, CA, USA) and the primers MD582F, MD1479RC, 12, and 3FL1 [9] to amplify fragments on the forward and reverse strands. Cycle sequence reactions contained 1 µL BigDye Terminator v3.1 Ready Reaction Mix, 1 µL 5x Sequencing Buffer, 3 µL of 1 µM primer, and 5 µL purified PCR product. Subsequently, cycle sequencing reactions were purified using Sephadex columns (Princeton Separation, Adelphia, NJ, USA) and centrifugation, followed by dehydration. Purified products were analysed on an ABI Prism 3730xl automated sequencer (Biotechnology Resource Center, Institute of Biotechnology, Cornell University, Ithaca, New York; Eurofins Genomics, Louisville, Kentucky). Raw sequence reads were proofed and chromatograms were visually inspected to verify all base changes using Sequencher 4.10.1 software (Gene Codes Corporation, Ann Arbor, Michigan).

### Characterization of PRNP exon 3

Nucleotide sequences were aligned and trimmed to the open reading frame of *PRNP* exon 3 using MEGA-X [33]. The trimmed nucleotide sequences were then translated into protein using the standard genetic code and assessed for any non-synonymous substitutions at the nucleotide and amino acid level. Additional nucleotide sequences of *PRNP* exon 3 representing CWD-susceptible taxa from Cervidae were obtained from GenBank and served as comparative sequences to ensure all substitutions, if any, were observed in axis deer.

### Phylogenetic analyses

To identify the phylogenetic placement of axis deer within Cervidae for *PRNP*, an initial dataset of 1,870 individuals were obtained from GenBank in addition to the 133 sequences from axis deer used in this study. *PRNP* sequences were obtained from GenBank for moose (n = 9), white-tailed deer (n = 1,149), mule deer (n = 589), reindeer/caribou (*R.* *tarandus*; n = 23), fallow deer (n = 5), elk (n = 44), red deer (n = 24), sika deer (n = 8), roe deer (n = 4), Pere David’s deer (*Elaphurus davidianus*; n = 1), Chinese water deer (*Hydropotes inermis*; n = 8), and Reeve’s muntjac (n = 6). RAxML version 8.2.12 [34] was used to identify identical sequences in this initial dataset and consequently, genotypes deemed identical (n = 1,848) were removed prior to subsequent phylogenetic analyses. A final dataset of 155 cervid *PRNP* sequences (length = 770 bp) was used in subsequent analyses. Sample sizes for each species in the subsequent analyses were as follows: axis deer: n = 1; moose: n = 5; white-tailed deer: n = 81; mule deer: n = 17; reindeer/caribou: n = 14; fallow deer: n = 1; elk: n = 8; red deer: n = 12; sika deer: n = 3; roe deer: n = 2; Pere David’s deer: n = 1; Chinese water deer: n = 6; and Reeve’s muntjac: n = 1.

Eighty-eight maximum likelihood models were evaluated using jModelTest-2.1.10 [35,36] and the Akaike Information Criterion with a correction for small sample sizes (AICc) [37] identified the K80+I+G model of evolution (-lnL = 2,326.0861) as the most appropriate for the dataset. A maximum likelihood analysis under a Bayesian inference model (MrBayes v3.2.6) [38] was conducted to generate posterior probability values (PPV). Reeve’s muntjac was selected as the outgroup based upon previously established Cervidae phylogeny [25]. The GTR+I+G (general time reversible plus inverse gamma) nucleotide substitution model and the following parameters were used: two independent runs with four Markov-chains (one cold and three heated; MCMCMC), 1.0 × 10^7^ generations, and sample frequency of every 1,000 generations from the last nine million generated. A visual inspection of likelihood scores resulted in the first 1,000,000 trees being discarded (10% burn-in) and a consensus tree (50% majority rule) constructed from the remaining trees. PPV ≥ 0.95 were used to designate nodal support [39].
